# Real‐time simulator for designing electron dual scattering foil systems

**DOI:** 10.1120/jacmp.v15i6.4849

**Published:** 2014-11-08

**Authors:** Robert L. Carver, Kenneth R. Hogstrom, Michael J. Price, Justin D. LeBlanc, Garret M. Pitcher

**Affiliations:** ^1^ Mary Bird Perkins Cancer Center Baton Rouge LA; ^2^ Department of Physics and Astronomy Louisiana State University Baton Rouge LA USA

**Keywords:** electron therapy, scattering foils, radiotherapy, analytical simulations, Monte Carlo

## Abstract

The purpose of this work was to develop a user friendly, accurate, real‐time computer simulator to facilitate the design of dual foil scattering systems for electron beams on radiotherapy accelerators. The simulator allows for a relatively quick, initial design that can be refined and verified with subsequent Monte Carlo (MC) calculations and measurements. The simulator also is a powerful educational tool. The simulator consists of an analytical algorithm for calculating electron fluence and X‐ray dose and a graphical user interface (GUI) C++ program. The algorithm predicts electron fluence using Fermi‐Eyges multiple Coulomb scattering theory with the reduced Gaussian formalism for scattering powers. The simulator also estimates central‐axis and off‐axis X‐ray dose arising from the dual foil system. Once the geometry of the accelerator is specified, the simulator allows the user to continuously vary primary scattering foil material and thickness, secondary scattering foil material and Gaussian shape (thickness and sigma), and beam energy. The off‐axis electron relative fluence or total dose profile and central‐axis X‐ray dose contamination are computed and displayed in real time. The simulator was validated by comparison of off‐axis electron relative fluence and X‐ray percent dose profiles with those calculated using EGSnrc MC. Over the energy range 7–20 MeV, using present foils on an Elekta radiotherapy accelerator, the simulator was able to reproduce MC profiles to within 2% out to 20 cm from the central axis. The central‐axis X‐ray percent dose predictions matched measured data to within 0.5%. The calculation time was approximately 100 ms using a single Intel 2.93 GHz processor, which allows for real‐time variation of foil geometrical parameters using slider bars. This work demonstrates how the user‐friendly GUI and real‐time nature of the simulator make it an effective educational tool for gaining a better understanding of the effects that various system parameters have on a relative dose profile. This work also demonstrates a method for using the simulator as a design tool for creating custom dual scattering foil systems in the clinical range of beam energies (6–20 MeV).

PACS number: 87.10.Ca

## I. INTRODUCTION

Due to characteristically high surface dose, relatively uniform dose plateau (surface to depth of 90% dose, $R_{90}$), and steep distal dose falloff with depth (90%–10% dose, $R_{90-10}$), electron beams can be utilized in radiotherapy to irradiate superficial targets while minimizing dose to underlying critical structures. Use of energies up to 20 MeV allows for the treatment of disease within approximately 6 cm of the surface while sparing deeper normal tissues.^(1)^

Hogstrom,^(1)^ Tapley,^(2)^ Vaeth and Meyer,^(3)^ and Gerbi et al.^(4)^ enumerate a number of patient sites for which electron beam therapy may be utilized. It is particularly useful in treatment of cancer of the skin such as eyelids, nose, ear, scalp, and lips, as well as more widely spread diseases of limbs (e.g., melanoma, lymphoma, and sarcoma) or total skin (mycosis fungoides). Electron beam therapy is also used to treat disease of the upper respiratory and digestive tract (e.g., floor of mouth, soft palate, retromolar trigone, and salivary glands), postmastectomy chest wall, postlumpectomy tumor bed, and lymph nodes. It is sometimes used to treat diseases of the retina or orbit, as well as spine (e.g., craniospinal irradiation) and paraspinal muscles. Also, electron beam therapy has been used intracavitarily and intraoperatively.

Currently, most linear accelerators employ a dual foil scattering system to achieve flatness for an electron beam, as proposed by Bjarngard et al.^(5)^ These dual foil systems are comprised of a primary thin metallic foil, typically high‐Z material, which scatters the initial narrow electron beam into an approximately Gaussian, off‐axis fluence profile 100 cm downstream at isocenter. The secondary foil, typically a conical shaped low‐Z material, which is located downstream of the primary foil, flattens the broadened beam. Designing a generic set of primary and secondary foils that cover a broad range of energies can be very complicated and inefficient, often involving a heavy reliance on time‐consuming measurements or Monte Carlo (MC) simulations that model the head of the linear accelerator. To improve design efficiency and for educational purposes, we developed an analytical dual scattering foil simulator with a user‐friendly graphical user interface (GUI).

The analytical calculation used for this study is an enhancement to the model presented by Green,^(6)^ which transported only the primary electron beam using Fermi‐Eyges theory.^7^, ^8^ Green utilized an energy and Z‐dependent correction factor to the ICRU 35‐calculated scattering powers. While this algorithm modeled neither electron scatter from the X‐ray jaws, electron scatter from the electron applicator, nor off‐axis X‐ray dose, it was able to accurately predict the Siemens‐measured data taken without collimation. Green's model was later improved by Kainz et al.,^(9)^ primarily by using the Gaussian term of the Molière method for calculating electron scattering powers. As with Green's model, this model was also able to reproduce MC calculations.^9^, ^10^

Our analytical algorithm further refined the scattering power calculation using the reduced‐Gaussian term from the Molière method. While Green used the most probable electron energy for the scatter power calculations, our model uses the mean electron energy. In addition, we refined the method for calculating the central‐axis X‐ray dose component so as to be applicable to a wider range of scattering foil systems. Finally, we added an off‐axis X‐ray relative dose simulation component which enables the calculation of off‐axis total relative dose profiles, in addition to off‐axis electron relative fluence profiles.

This study demonstrated the accuracy and versatility of our real‐time off‐axis total relative dose and electron relative fluence profile dual scattering foil simulator. It also provided examples of how this simulator can be used for design applications for electron dual scattering foil systems, as well as educational purposes. The real‐time feedback nature of the simulator makes it a useful and efficient tool for both engineering design and education.

## II. MATERIALS AND METHODS

### A. Analytical simulator

#### A.1 Modeling electron dose component

**Figure 1:**
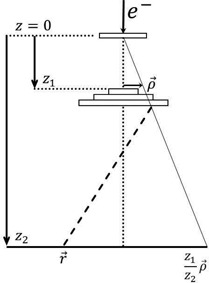
Illustration depicting the dual scattering foil geometry and variables used by the analytical simulation algorithm.

The geometry of the dual foil scattering system is illustrated in Fig. 1. The central z‐axis corresponds to the direction of the incident electron beam. The top of the primary foil, the top of the secondary foil, and the calculation plane are located at $z=0,\;z_{1}$, and $z_{2}$, respectively. The vector $\vec{\rho }$ corresponds to the radial position of a given pencil beam at $z_{1}$. The mean directions of the pencil beams at $z_{1}$ emanate from the primary foil such that the position $\vec{\rho }$ at $z_{1}$ projects to $\frac{z_{2}}{z_{1}}\;\vec{\rho }$ at $z_{2}$

##### A.1.1 Modeling electron fluence

The electron planar fluence at $z_{2}$ per incident electron, contained in Kainz et al.,^(9)^ is given by:

(1) $$\Phi_{e}(z_{2},\vec{r},E)=\int_{0}^{2\pi }\int_{0}^{\rho_{\text{max}}}\Phi_{e}(z_{1},\vec{\rho },E)\;\Phi_{e}(z_{2}-z_{1},\vec{r},\vec{\rho },E)\rho d\rho d\Theta ,$$

where $\Phi_{e}(z_{2},\;\overset{⇀}{r},\;E)$ is the convolution of $\Phi_{e}(z_{1},\;\overset{⇀}{\rho },\;E)$, the electron planar fluence distribution at $z_{1}$ per incident electron, and $\Phi_{e}(z_{2}–z_{1},\overset{⇀}{r},\overset{⇀}{\rho },E)$, the planar fluence at location $\overset{⇀}{r}$, in the plane $z_{2}$, per incident electron at location $\vec{\rho }$ in the plane $z_{1}$. The off‐axis electron relative fluence profile in air equals the off‐axis electron relative dose profile in water at shallow depths due to the assumption of a constant energy spectrum versus off‐axis position in our model. A more complete description of the algorithm can be found in Kainz et al.^(9)^

A few changes were made to improve the accuracy of the algorithm described by Kainz and colleagues. The Molière Gaussian approximation for scattering power was replaced with the reduced Gaussian approximation ^(11)^ $\theta_{\text{Reduced}\;\text{Gaussian}}^{2}=\theta_{\text{Moli}\overset{`}{e}\text{re}\;\text{Gaussian}}^{2}(1–\frac{1.330}{B})$, where *B* is related to the number of collisions encountered by an electron propagating through the scatterer and is defined in Kainz et al.^(9)^ In addition, the Kainz study used the most probable energy to calculate the scattering power; however, we found that using the most probable energy and the reduced Gaussian method underestimated the observed scatter. As a beam passes through energy degraders (i.e., the vacuum exit window and scattering foils) the low energy tail of the energy distribution begins to grow and, using the most probable energy, underestimates the effects of this low energy tail. In order to better account for the low energy tail, we used the beam's mean energy when calculating scattering powers.

To determine the mean energy incident on each beam modifying element, the total energy loss (radiative and collisional) due to each element was determined using electron stopping powers calculated from ICRU Report 35 (Eqs. 2.2 and 2.4).^(11)^ The mean energy loss through the vacuum window and primary foil was calculated by multiplying the total (radiative and collisional) linear stopping power by the appropriate thickness. For calculating the scattering power of the secondary foil at each integration point $\vec{\rho }$, the average of the mean incident and exiting energy was used. The mean exiting energy over the entire exiting beam was calculated by weighting the energy loss through the secondary foil at each integration point by the incident relative electron fluence.

##### A.1.2 Electron fluence to dose conversion

Assuming the electron dose component $\left(D_{e}\right)$ is proportional to the electron fluence (i.e., assuming the mass stopping power in water varies insignificantly with energy in the range of interest; Note: from 4 MeV, the energy of a 6 MeV beam at 1 cm depth, to 16 MeV, the energy of a 20 MeV beam at 2 cm depth, it falls in the range of $1.94\pm 0.08\;{\text{MeVg}}^{-1}{\text{cm}}^{2}$), then:

(2) $$D_{e}(d,r,E)=K_{e}(E_{d})\phi_{e}(d,r,E).$$

$K_{e}(E_{d})$ is the electron collisional mass stopping power at $E_{d}=E_{p,0}–d\;*\;2\;\text{MeV}/\text{cm},\;E_{p,0}$ is the most probable energy at the surface, $d=z_{2}-\text{SAD}$, and $\phi_{e}$ is the electron fluence from Eq. (1). *SAD*, the source‐to‐axis (isocenter) distance, is 100 cm.

#### A.2 Modeling X‐ray dose component

The simulator utilizes X‐ray dose calculations to provide two key items: 1) the central‐axis X‐ray dose (as a percentage of the central‐axis dose maximum) at a depth of $R_{p}+2\;\text{cm},\;\%D_{\gamma ,\text{CAX}}(R_{p}+2\text{cm},\;E)$, and 2) the off‐axis distribution of total relative dose, $D_{T}(d,\;r,\;E)$, at depth *d* in a water phantom whose surface is at isocenter.

Paralleling the derivation by Schiff,^(12)^ which is based on multiple Coulomb scattering theory, the number of X‐rays $\left(\Delta N_{\gamma }\right)$ emitted per unit solid angle (ΔΩ) with respect to the direction of the incident electron beam of energy *E* is given by:

(3) $$\begin{array}{l} \;\;\;\;\;\frac{\Delta N_{\gamma }}{\Delta \Omega }(\theta_{r},E)=\\ K_{\gamma }\sum_{i=1}^{3}\frac{\Delta E_{\text{rad},i}(E)}{\pi T_{i}(E)t_{i}}\left[E_{1}\left(\frac{\theta_{r}^{2}}{A_{0,i}(E)+T_{i}(E)t_{i}}\right)-E_{1}\left(\frac{\theta_{r}^{2}}{A_{0,i}(E)}\right)\right], \end{array}$$

where $\theta_{r}$ is the radial angle from the central axis, $\Delta E_{\text{rad},i}(E)$ is the radiative energy loss from the *ith* scattering element for the beam energy $E,T_{i}(E)$ is the energy dependent linear scattering power of the *ith* scattering element, $t_{i}$ is the mean thickness of the *ith* scattering element, $E_{1}$ is the exponential integral function, $K_{\gamma }$ is a constant of proportionality, and $A_{0,i}$ is the zeroth‐order scattering moment of the electron entering the *ith* scattering element given by:

(4) $$A_{0,1}(E)=A_{0,i-1}(E)+T_{i-1}(E)t_{i-1}.$$

In theory, $A_{0,0}$ is the zeroth‐order scattering moment of the electron beam incident on the first scattering element, the accelerator vacuum window. The primary and secondary foils correspond to i=2 and 3, respectively. The X‐ray fluence $\left(\Delta N_{\gamma }/\Delta A\right)$ at an off‐axis distance *r* and at a distance *z* from the virtual source ($r=z\;\theta_{r}$ and $\Delta A=z^{2}\Delta \Omega$) is given by:

(5) $$\begin{array}{l} \;\;\;\;\;\phi_{\gamma }(z,r,E)=\\ \frac{K_{\gamma }}{z^{2}}\sum_{i=1}^{3}\frac{\Delta E_{\text{rad},i}(E)}{T_{i}(E)t_{i}}\left[E_{1}\left(\frac{{\left(\frac{r}{z}\right)}^{2}}{A_{0,i}(E)+T_{i}(E)t_{i}}\right)-E_{1}\left(\frac{{\left(\frac{r}{z}\right)}^{2}}{A_{0,i}(E)}\right)\right], \end{array}$$

where the constant π has been absorbed into $K_{\gamma }$.

Assuming the resulting X‐ray dose is proportional to $\phi_{\gamma }$ (i.e., assuming the mass energy absorption coefficient in water varies insignificantly with photon energy in the range of interest; Note: from 2 to 6 MeV, mean energies of 6 to 18 MV X‐ray beams, it falls in the range of $0.022\pm 0.004\;{\text{cm}}^{2}g^{-1}$), then for $K\prime_{\gamma }(E)$ equaling $K_{\gamma }$ times the mass energy absorption coefficient in water:

(6) $$\begin{array}{l} \;\;\;\;\;D_{\gamma }(z,r,E)=\\ \frac{K_{\gamma }^{'}(E)}{z^{2}}\sum_{i=1}^{3}\frac{\Delta E_{\text{rad},i}(E)}{T_{i}(E)t_{i}}\left[E_{1}\left(\frac{{\left(\frac{r}{z}\right)}^{2}}{A_{0,i}(E)+T_{i}(E)t_{i}}\right)-E_{1}\left(\frac{{\left(\frac{r}{z}\right)}^{2}}{A_{0,i}(E)}\right)\right], \end{array}$$

The evaluation of Eq. (6) is complicated by the properties of the $E_{1}$ function at r=0. Evaluating the terms in brackets from Eq. (6) using a series expansion yields

(7) $$\underset{r\to 0}{\text{lim}}\left[E_{1}\left(\frac{{\left(\frac{r}{2}\right)}^{2}}{A_{0,i}+T_{i}t_{i}}\right)-E_{1}\left(\frac{{\left(\frac{r}{2}\right)}^{2}}{A_{0,i}}\right)\right]=\text{ln}\left(1+\frac{T_{i}t_{i}}{A_{0,i}}\right).$$

In the evaluation of the $E_{1}$ terms in Eq. (6), using Eq. (7), the $E_{1}$ function goes to ∞ if $A_{0,0}=0$. This is a result of our assuming the X‐ray dose is exactly proportional to the X‐ray fluence, as opposed to convolving $\phi_{\gamma }$ with the forward‐peaked bremsstrahlung distribution (very much like a delta function being proportional to $(\text{sin}\;{\theta_{r})}^{-4}$). Empirically, we found that $A_{0,0}=0.05E^{-1.6}$ for the incident electron beam energy *E* in MeV resulted in the best fit of analytical X‐ray fluence versus off‐axis position (*r*) to MC‐simulated profiles for electron beams of normal incidence from 6–20 MeV

##### A.2.1 Modeling central‐axis X‐ray dose

In the present work, central‐axis X‐ray percent dose was expressed as a percent of central‐axis dose maximum at a depth of $R_{p}+2\;\text{cm}$ with the water phantom surface at isocenter (i.e., $\%D_{\gamma ,\text{CAX}}(R_{p}+2,E)$). Assuming that the central‐axis electron dose maximum $\left(d\approx R_{100}\right)$ is proportional to the central‐axis electron fluence at the surface (d=0) and that the central‐axis X‐ray dose component is much less than the electron dose component $\left(D_{\gamma }(R_{100})\;«\;D_{e}(R_{100})\right)$, the central‐axis X‐ray percent dose $\left(d=R_{p}+2\;\text{cm}\right)$ can be approximated by:

(8) $$\%D_{\gamma ,\text{CAX}}(R_{p}+2,E)=\frac{D_{\gamma ,\text{CAX}}(R_{p}+2)}{D_{\gamma ,\text{CAX}}(R_{100})+D_{e,\text{CAX}}(R_{100})}\approx \frac{D_{\gamma ,\text{CAX}}(R_{p}+2,E)}{D_{e,\text{CAX}}(R_{100},E)}.$$

Then, using Eqs. (2), (6), (7):

(9a) $$\%D_{\gamma ,\text{CAX}}(R_{p}+2,E)=K^{\prime }X(E)$$

where

(9b) $$X(E)=\frac{1}{{\left(\text{SAD}+R_{p}+2\right)}^{2}}\left[\frac{\sum_{i=1}^{3}\frac{\Delta E_{\text{rad},i}}{T_{i}t_{i}}\text{ln}\left(1+\frac{T_{i}t_{i}}{A_{0,i}}\right)}{\phi_{e}(0,0,E)}\right],$$

and

(9c) $$K^{\prime }=K_{\gamma }^{'}/K_{e}.$$

While $K\prime_{\gamma }$ and $K_{e}$ depend on the mass absorption coefficient and collisional mass stopping power, respectively, they are weakly varying with energy over the clinical range of electron beams. Thus, K′ was treated as energy independent for the purposes of this model. According to Eq. (9a) a plot of the $\%D_{\gamma ,\text{CAX}}(R_{p}+2,E)$ versus *X(E)* should be a straight line passing through the origin with a slope of K′. However, a linear fit to MC calculations for multiple beams with different E, primary foils, and secondary foils showed a y‐intercept of 1.0% and an error of up to 1.0%. An exponential term was added in order to force the fit through the origin and to allow for the slight nonlinear dependence of K′ (i.e., the data were fit to):

(10) $$\%D_{\gamma ,\text{CAX}}(R_{p}+2,E)=\text{aX}(E)+b(1-e^{-\text{cX}(E)}).$$

**Figure 2:**
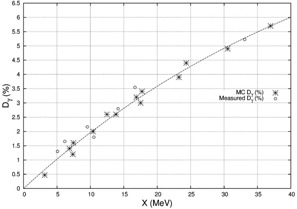
Plot showing MC‐calculated (stars) and measured (circles) central‐axis X‐ray percent doses at $d=R_{p}+2\;\text{cm}$ vs. the term *X*, as defined in Eq. (9b). Dashed line represents the result of fitting Eq. (10) to the MC‐calculated data.

As shown in Fig. 2, the best fit showed $a=-0.035\;\%\;\text{Me}V^{-1}$, b=15.77%, and $c=0.016\;\text{Me}V^{-1}$ with fit and MC‐calculated values agreeing to within ±0.5%.

##### A.2.2 Modeling off‐axis X‐ray dose

The off‐axis X‐ray percent dose profile at a depth *d* was the product of the central‐axis X‐ray percent dose and the off‐axis X‐ray relative dose profile. The off‐axis X‐ray relative dose profile was calculated from Eq. (6) by taking ratio $D_{\gamma }(z,\;r,\;E)\;/\;D_{\gamma }(z,\;0,\;E)$, where Eq. (7) was used to calculate the dose at r=0, resulting in

(11) $$\%D_{\gamma }(d,r,E)=\left[\frac{D_{\gamma }(\text{SAD}+d,r,E)}{D_{\gamma }(\text{SAD}+d,0,E)}\right]\%D_{\gamma ,\text{CAX}}(d,E),$$

where the central‐axis X‐ray percent dose was calculated from Eq. (10) and corrected for the inverse square to depth *d*:

(12) $$\%D_{\gamma ,\text{CAX}}(d,E)=\%D_{\gamma ,\text{CAX}}(R_{p}+2,E){\left(\frac{\text{SAD}+R_{p}+2}{\text{SAD}+d}\right)}^{2}$$

(Note: X‐ray attenuation was approximately 9%±2% at all energies and was ignored.)

Despite multiple approximations, by tying the semi‐empirical calculations based on multiple Coulomb scattering theory to MC‐calculated values of central‐axis X‐ray percent dose and off‐axis X‐ray relative fluence profiles, the simulator was sufficiently accurate for modeling X‐ray dose in electron beams. This accuracy is validated in the results.

#### A.3 Modeling total dose

The off‐axis total relative dose profile, normalized to 100% at r=0, was calculated by combining the off‐axis X‐ray percent dose and off‐axis electron relative dose profiles:

(13) $$D_{T}(d,r,E)=\%D_{\gamma }(d,r,E)+[100\%-\%D_{\gamma ,\text{CAX}}(d,E)]\frac{D_{e}(d,r,E)}{D_{e}(d,0,E)}.$$

**Figure 3:**
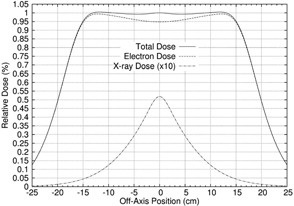
Plot of calculated off‐axis relative dose profiles for the electron, X‐ray, and total dose components for a sample 20 MeV beam at depth of 2 cm. The X‐ray dose component (5.2% at the calculation depth) has been magnified by a factor of 10 to make it more easily visible.

Figure 3 compares the calculated off‐axis electron, X‐ray, and total relative dose profiles for a 20 MeV beam at a depth of 2 cm. The difference in the shape of the off‐axis total and electron relative dose profiles illustrates the need to include the off‐axis X‐ray percent dose profile in designing a dual scattering foil system, particularly at the higher energies, where $\%D_{\gamma ,\text{CAX}}(R_{p}+2,E)$ is greater.

### B. Validation of simulator using BEAMnrc Monte Carlo

The analytical simulator's ability to accurately calculate dose profiles was validated using MC models based on the geometry of an Elekta Infinity accelerator (Elekta AB, Stockholm, Sweden). The simulator's electron dose calculations were validated for the primary foil and dual foil configurations. Also, the magnitudes and shapes of the X‐ray dose component were validated.

The MC model used for validation of the analytical simulator has been previously validated against measured data for an Elekta Infinity accelerator. A complete description of the MC validation can be found in the Master's thesis of G.M. Harris.^(13)^ In addition, comparisons of large field measured off‐axis electron relative dose profiles $\left(40\times 40\;{\text{cm}}^{2}\right)$ with analytical simulations can be found in the Master's thesis of J.D. LeBlanc.^(14)^

#### B.1 Validation of simulator primary foil calculations

**Figure 4:**
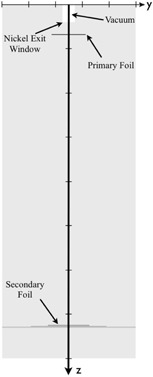
Schematic depicting the accelerator geometry used in the primary and secondary scattering foil validation of the real‐time dual scattering foil simulator. For simulations of the primary scattering foil only, the secondary scattering foil was removed from the geometry.

These validations consisted of comparisons between MC and analytical simulator calculations containing only a 0.0125 cm thick nickel exit window (vacuum to air) and appropriate tantalum primary foil thickness for the energy being studied (Fig. 4). Without the secondary foil, the analytical simulator predicted the electron fluence profile (excluding X‐ray dose) as a Gaussian at the calculation plane (z=101cm for $E_{p,0}<9\;\text{MeV}$ and z=102cm for $E_{p,0}\geq 9\;\text{MeV}$). MC simulations provided a method by which to assess the accuracy of this model. In order to make the MC parameters congruent with the calculations of the analytical simulator for these tests, the incident electron beam was modeled with zero angular spread, zero radial spread, and the incident energy being monoenergetic. The MC dose and fluence distributions were calculated and stored using voxels that were $5\times 5\times 5\;{\text{mm}}^{3}$. A total of 2 billion histories ensured adequate statistical precision (≈1%) at each voxel of the dose grid. From the resulting phase spaces file, the off‐axis electron planar fluence profiles (z=101cm for $E_{p,0}<9\;\text{MeV}$ and z=102cm for $E_{p,0}\geq 9\;\text{MeV}$) were extracted and normalized to 100% on central axis for comparison with results of analytical simulations. Comparisons were performed for the range of seven energies (7–20 MeV) offered by the Elekta Infinity accelerator (S/N 151892) at Mary Bird Perkins Cancer Center (MBPCC) in Baton Rouge, LA. The primary foil's tantalum thicknesses ranged from approximately 70 μm to 140 μm.

#### B.2 Validation of simulator dual foil calculations

A more rigorous validation of the overall analytical simulator was performed, which consisted of including only the exit window, primary foil, and secondary foil in both the MC and analytical simulator calculations (Fig. 4). For the analytical simulator calculations, the Elekta secondary scattering foil geometries were input using data files containing their shape and material composition. The same Elekta secondary scattering foil geometries were used in MC simulations of 2 billion histories, ensuring adequate statistical precision (≈1%). From the resulting phase space files, the off‐axis electron planar fluence profiles (z=101cm for $E_{p,0}<9\;\text{MeV}$ and z=102cm for $E_{p,0}\geq 9\;\text{MeV}$) were extracted and normalized to 100% on the central axis for comparison with the result of analytical simulations. Agreement between the MC and analytical simulator calculations were evaluated across the full range of the MC data (±24.5cm), although the region of interest for this study was that of ±17.6cm from the central axis, the distance corresponding to the span of the diagonal of a $25\times 25\;{\text{cm}}^{2}$ field, the size of the largest applicator.

#### B.3 Validation of simulator X‐ray dose calculations

The analytical simulator's calculation of central‐axis X‐ray percent dose $\%D_{\gamma ,\text{CAX}}(R_{p}+2,E)$, defined as the dose at a depth of $R_{p}+2\;\text{cm}$, was validated by comparison to measured clinical data. Agreement between the two would provide a high degree of confidence that the analytical simulator can accurately predict X‐ray dose for cases with no measured data. Measured $\%D_{\gamma ,\text{CAX}}$ values were taken from clinical commissioning data for the MBPCC Elekta Infinity accelerator. Clinical data had been acquired in accordance with TG‐25^(15)^ and TG‐70^(4)^ protocols.

Also, a validation of the analytical calculation of off‐axis X‐ray percent dose profiles was conducted by comparison with MC calculated profiles at shallow depths. The MC simulation contained only components included in the analytical simulator (i.e., exit window, primary foil, and secondary foil). Comparisons were done over the range of energies available for the Elekta Infinity accelerator (7–20 MeV).

#### B.4 BEAMnrc description

The EGSnrc MC package is an electron and photon transport modeling utility that contains the physics necessary to simulate electron and X‐ray beams generated by radiotherapy electron accelerators.^(16)^ BEAMnrc, which is built on EGSnrc, is accompanied by precoded component modules, thereby making the geometric construction of an accelerator significantly simpler. The BEAMnrc model for the Elekta accelerator used in the current work was developed by Harris.^(13)^ Geometrically, the inputs to the EGSnrc model consisted of all relevant machine components, which included exit window, primary foil, primary collimator, secondary foil, ion chamber, mirror (for optical distance indicator), X‐ray collimation (jaws and multileaf collimator), electron applicator, and collimating insert. Harris determined beam characteristics, including incident (z=0) Gaussian energy spectra, initial angular divergence of the beam (0°), and Gaussian focal spot size (2mm×1mm FWHM) that resulted in a good match with clinical data. The identical input files were used in the present study, with the exception of spot size for which 1mm×1mm FWHM was used so as to maintain X‐Y symmetry in the model. Global cutoff energies of 0.521 MeV and 0.01 MeV were used for electrons and photons, respectively.

EGSnrc can generate phase spaces at desired spatial locations. These phase spaces contain relevant information about radiation transported by the code, such as particle type, energy, position, and direction. For the validation of the analytical simulator, phase spaces were scored in air at 101 cm for the beam energy of 7 MeV and at 102 cm for all beam energies 9 MeV and greater. Phase spaces were then analyzed using BEAMDP, which is a component program of the EGSnrc software package. BEAMDP allows for the extraction of phase spaces' electron planar fluence profiles, which were then directly comparable to the analytical simulator's output, once normalized.

All MC simulations were calculated using Tezpur, one of Louisiana State University's High‐Performance Computing (HPC) clusters (http://www.hpc.lsu.edu/). Tezpur runs Red Hat Enterprise Linux 4 and is comprised of 360 compute nodes, each containing two 2.66GHz Dual Core Xeon 64‐bit processors.

### C. GUI for analytical calculations of dual scattering foil system

The electron planar fluence and X‐ray dose calculation algorithms were incorporated into a user friendly GUI. From this GUI, the user has the ability to change the essential calculation parameters. Most relevant to this study are beam energy and parameters dealing with the primary and secondary foil characteristics, such as material composition, central‐axis thickness, and shape of the secondary foil. While the material composition is limited to a single element, it is possible to simulate a compound by using effective atomic number and weight.

#### C.1 Primary foil options

**Figure 5:**
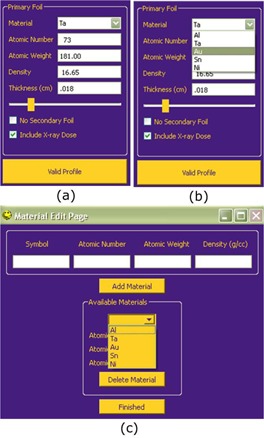
Screenshots of (a) the analytical simulator's GUI primary foil parameter pane, (b) the material dropdown menu, and (c) the window allowing the user to add or delete materials available for simulation. By checking “No Secondary Foil”, results for only the primary foil are calculated. By checking “Include X‐ray Dose”, off‐axis X‐ray percent dose is added to the electron dose for the simulated total dose profile.

The primary foil pane, shown in Figs. 5(a) and 5(b), enables the user to specify the material composition and thickness of the primary foil. The “Material” dropdown box (Fig. 5(b)) allows the user to select from a set of user‐defined compositions for the primary foil. New materials can be added by the user via a separate window (Fig. 5(c)). The thickness can be varied by manually entering a thickness or by using the slider bar. The effects of changing these parameters on the resulting off‐axis total relative dose profile and central‐axis X‐ray percent dose are displayed in real time. Additionally, in the primary foil pane, the user has the option to calculate the off‐axis profile with the secondary foil absent and with or without the X‐ray dose included.

#### C.2 Secondary foil options

**Figure 6:**
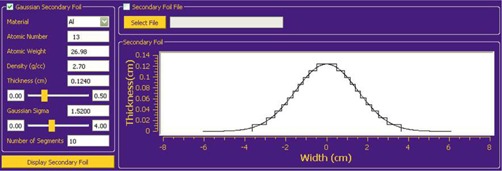
Screenshot of the analytical simulator's GUI secondary foil parameter pane. The “Gaussian Secondary Foil” panel on the left contains the parameters and slider bars for the thickness and sigma of a Gaussian‐shaped, user‐defined secondary foil. The “Secondary Foil” panel displays the cross‐sectional shape of the secondary foil Gaussian and its segmented approximation. Note that the thickness and width axes are scaled differently.

The secondary foil pane (Fig. 6) has more design options due to the secondary foil's increased complexity. The user has the option to select a secondary foil data file in which the foil's material composition and geometry are specified. In this way, the user is able to input a pre‐existing or new non‐Gaussian foil design. Alternatively, the user has the option to select a foil design based on a Gaussian shape. In this case, the user specifies the material (typically aluminum), central‐axis thickness of the foil, the sigma of the Gaussian‐shaped cross section, and the number of equally spaced foil segments with which to approximate the Gaussian shape. The latter option models how a secondary foil is typically manufactured. The analytical simulator then determines the thickness of each segment based on the number of segments specified, and it approximates the shape of the Gaussian by intersecting midpoint of the descending edge of each segment with that of the true Gaussian. Both the thickness and the sigma of the Gaussian secondary foil can be varied by manual input or by slider bars, and the resulting secondary foil drawing and the predicted dose distributions (total or electron only) are displayed in real time. This capability allows for rapid examination of multiple foil parameters, as required for optimizing foil design.

#### C.3 Simulator geometry and calculation options

To define the simulator geometry of the accelerator, the user chooses the “Simulation Settings” page from the “Tools” menu. This page allows the user to set the distances from the vacuum window to primary foil, primary foil to secondary foil, secondary foil to isocenter, and isocenter to calculation plane (calculation depth). The user can also define the beam energy as either the monochromatic energy incident on the accelerator vacuum window or as the most probable energy at isocenter, $E_{p,0}$. In addition, this page allows user to set calculation options such as the lateral extent of the calculated profile (30 cm), number of integration points in radius and theta space (100), number of profile points calculated (60), and maximum radius of the secondary foil (3.65 cm), with typical values shown in parentheses. Increasing values of the calculation options (parameters) will increase the off‐axis relative profile calculation times. The user can also set the flatness criteria and off‐axis range over which to apply the flatness criteria. Most of these options are kept constant during the optimization or evaluation of a dual scattering foil system for an accelerator and, thus, are not available for modification on the main screen.

**Figure 7:**
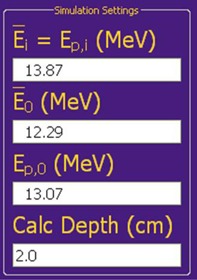
Screenshot of the analytical simulator's GUI simulations settings pane. The calculation depth and beam energy can be modified in this pane. If the forward energy calculation option is selected then the accelerator energy, ${\bar{E}}_{i}$, is entered and the simulator calculates the energy parameters ($E_{p,0}$ and ${\bar{E}}_{0}$) at the surface (isocenter). If the forward energy calculation option is not selected, then the user enters the most probable energy at the surface $\left(E_{p,0}\right)$, and the simulator calculates the initial most probable monochromatic energy. Note that the most probable energy is only displayed to help relate simulations to clinical surface energies $\left(E_{p,0}\right)$, but is not used for any scattering or stopping power calculations.

The geometry and calculation options most often modified — the calculation depth and beam energy — are available on the simulation settings pane (Fig. 7) of the main screen. The calculation depth is defined as the distance past isocenter where the in‐air profile is calculated (i.e., scatter in water is insignificant and ignored). Increasing the calculation depth increases the width of the simulated profile by essentially projecting its shape. Since at shallows depths it is assumed that the off‐axis electron relative fluence in air is equal to the off‐axis electron relative dose in water, calculation depths should be kept under 2 cm.

#### C.4 Visualization options

**Figure 8:**
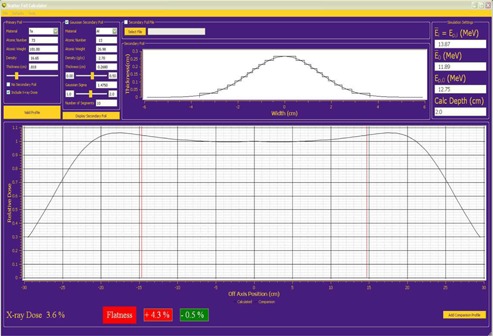
Screenshot of the analytical simulator GUI showing the total relative dose profile for a sample dual scattering foil. At the bottom left of the pane, the X‐ray dose is given as a percent of the central‐axis dose maximum. Using a horizontal line at 1.0 as the objective profile, the flatness was evaluated and the maximum deviations above and below the objective profile are displayed. The red vertical lines represent the lateral extent (±14.6cm) over which the flatness criteria were evaluated. The minimum flatness measure appears in green to indicate that this design passed a user‐defined criterion (in this case within 3%) below 100%, while the maximum flatness measure appears in red to indicate that this design was outside the criterion above 100%.

The profile pane, illustrated in Fig. 8, shows the real‐time display of the off‐axis relative dose profile and the central‐axis X‐ray percent dose for a given dual scattering foil combination. Also, the user has the option to display a comparison profile along with the calculated profile by using the “Add Comparison Profile” button and selecting a file with a proper format (position, relative dose). If this option is chosen, the analytical simulator automatically calculates the extremes of the deviation between the two profiles within a user‐specified range and compares it to user‐defined flatness criteria. This flatness evaluation displays the maximum deviation above and below the comparison profile, using the display color red to indicate failing and green to indicate passing.

#### C.5 Computational time

One of the major advantages of the analytical simulator is its computational time as compared to the MC simulations. The analytical simulation (0.5 cm spatial resolution) takes approximately 100 ms on a typical desktop personal computer, or approximately $3\times 10^{-5}$ processor hrs. Contrastingly, computational times for the MC simulations of beam lines composed of both primary and secondary foils were approximately 5–6 hrs when using 80 processors on Tezpur (i.e., between 400 and 480 processor hrs). Thus, the analytical simulator was approximately 10^7^ times faster than our typical MC simulation.

## III. RESULTS & DISCUSSION

### A. Validation of analytical simulator calculations

The analytical simulator was validated by comparing analytical and MC‐calculated off‐axis electron fluence profiles with geometries containing only the primary scattering foil and those containing both the primary and secondary scattering foils. In addition, the analytical and MC‐calculated central‐axis X‐ray percent dose at a depth of $R_{p}+2\;\text{cm}$ and off‐axis X‐ray percent dose profiles were compared. As mentioned in the Methods section, the MC model used for these comparisons was previously validated by Harris.^(13)^

#### A.1 Off‐axis primary foil validation

**Figure 9:**
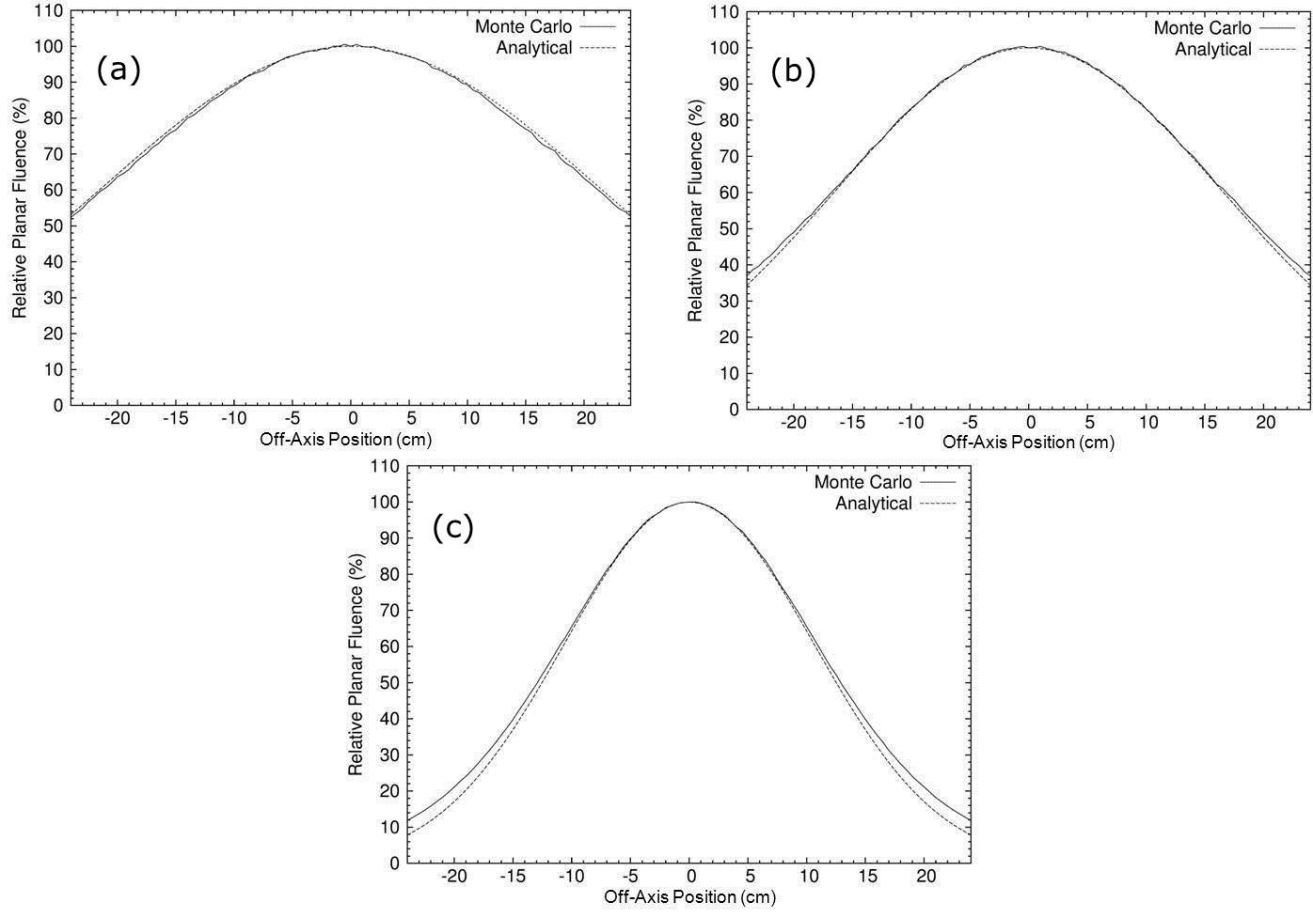
Comparisons of electron relative planar fluence profiles calculated by the analytical simulator with that calculated by MC simulations of primary foil only geometry for (a) 7 MeV, (b) 13 MeV, and (c) 20 MeV electron beams. Plots are 1 cm, 2 cm, and 2 cm downstream of isocenter, respectively.

Analytical simulations of the primary foil only geometry produced off‐axis electron relative planar fluence profiles that agreed well with those from MC simulations across the energy range of the Elekta Infinity accelerator (7–20 MeV). Figure 9 shows the agreement at three energies (7, 13, and 20 MeV); for all energies within the off‐axis range (−24cm, 24 cm) the greatest deviation from the MC simulations was 4.1% at 20 MeV. Table 1 shows the nominal beam energy, monochromatic energy incident on the exit window, average energy at isocenter, and most probable beam energy at isocenter. For each beam energy, it shows the maximum deviation of the off‐axis electron relative planar fluence from the MC simulation, with the higher energies showing greater deviations. These deviations are likely a result of the reduced Gaussian scattering approximation used by the analytical simulator for calculating the fluence profile. The MC simulation used the full Molière scattering theory, which results in a profile that is not a simple Gaussian, particularly at the large angles. This large angle scatter only was evident in the higher energy simulations because, at lower energies, the spread caused by the primary foil was such that the large angle effects occurred well outside the simulated off‐axis range (−24cm, 24 cm).

**Table 1:** Maximum differences between the MC and analytical simulations of off‐axis electron relative planar fluence profiles within the off‐axis range (−24cm, 24 cm) for a simulation geometry including the primary foil only; differences are expressed as a percent of central‐axis fluence. Also listed are differences between the measured and analytical central‐axis X‐ray percent dose at a depth of $R_{p}+2\;\text{cm}$; differences are expressed as a percent of central‐axis dose maximum. $E_{\text{nom}}$ are the nominal beam energies for our Elekta accelerator. ${\bar{E}}_{0}$ and $E_{p,0}$ are the corresponding average and most probable energies, respectively, measured at isocenter. $E_{p,i}$, the most probable energies incident on the nickel exit window, were calculated by the analytic simulator. For this study the beams incident on the nickel foil were assumed monoenergetic; hence, the incident mean energies equaled incident most probable energies.

$E_{\text{nom}}$ *(MeV)*	$E_{p,i}={\bar{E}}_{i}$ *(MeV)*	${\bar{E}}_{0}$ *(MeV)*	$E_{p,0}$ *(MeV)*	*Off‐Axis Electron Fluence Profile Maximum Difference (MC‐Analytical)*	*Central‐axis X‐ray Dose Difference (Measured – Analytical)*
7	7.87	6.95	7.15	1.6%	0.2%
9	9.53	8.26	8.66	2.5%	0.3%
10	10.62	9.63	9.92	3.2%	0.3%
11	12.06	10.83	11.28	2.6%	0.3%
13	13.99	12.46	13.13	2.7%	0.2%
16	17.44	15.50	16.22	4.4%	0.5%
20	21.80	19.09	20.50	4.1%	−0.1%

#### A.2 Off‐axis primary and secondary foil validation

**Figure 10:**
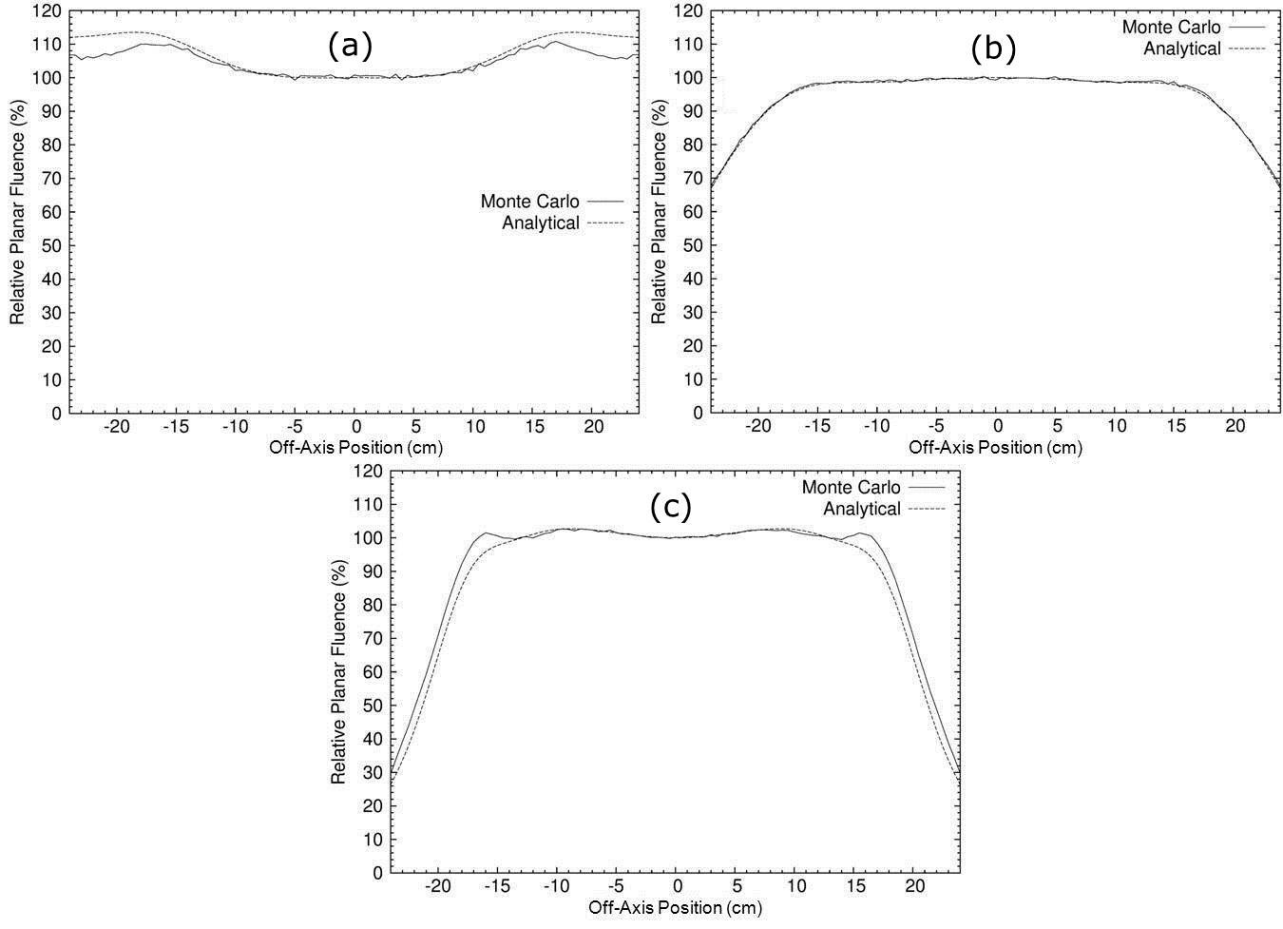
Comparisons of electron relative planar fluence profiles calculated by the analytical simulator with that calculated by MC simulations of primary and secondary foil geometry for (a) 9 MeV, (b) 11 MeV, and (c) 20 MeV electron beams. Plots are 2 cm downstream of isocenter.

Comparisons of the off‐axis electron relative planar fluence profiles calculated by analytical and MC simulations in the presence of both the primary and secondary foils for an Elekta accelerator showed varying agreement. For all energies, electron relative planar fluence from the analytical and MC simulations agreed well within 10 cm of the central axis, with the largest deviation being 2.1% for 9 MeV beam. For the lowest energies, 7 and 9 MeV, the analytical simulator consistently overpredicted that from the MC simulations by a maximum of 3.5% over the off‐axis range (−17.6cm, 17.6 cm). Figure 10(a) shows the comparison of the electron relative planar fluence from MC and analytical simulations for the 9 MeV beam, which demonstrates the case of worst agreement.

For the energies of 10, 11, and 13 MeV, the electron relative planar fluence calculated by the analytical and MC simulations agreed extremely well. Over the entire off‐axis range of the MC data (−24cm, 24 cm), the greatest deviation in the electron relative planar fluence calculated by the two methods was 2.4%. Within the off‐axis range (−17.6cm, 17.6 cm), the maximum deviation was 1.6%. Figure 10(b) shows the agreement for the 11 MeV beam, which is representative of the agreement within this energy range.

Figure 10(c) compares the electron relative planar fluence calculated by the analytical and MC simulations for the 20 MeV beam. As expected from the results of the primary foil only simulations, the agreement for the highest energy beams, 16 and 20 MeV, showed poorer agreement at the larger off‐axis positions. These large angle deviations were again likely caused by the reduced Gaussian approximation used for determining fluence profiles. However, within the off‐axis range (−12cm, 12 cm) where the Gaussian approximation for the primary fluence was valid, the maximum deviation was 1.3%.

#### A.3 X‐ray dose validation

The analytical simulator's calculation of central‐axis X‐ray percent doses at $R_{p}+2\;\text{cm}$ for the Elekta accelerator agreed well with measured values. Figure 2 plots the central‐axis X‐ray percent doses, both measured and MC calculated, versus the calculated variable *X* as defined in Eq. (9b). The equation of the dashed line, the least square fit of Eq. (10) to the MC‐calculated X‐ray doses versus *X*, is the function incorporated into the analytical simulator. The X‐ray dose differences (measured ‐ predicted) for the 7 MeV to 20 MeV beams are listed in Table 1, and the greatest deviation of the analytical prediction from the measured data, which occurred for the 16 MeV beam, was 0.5% of the central‐axis dose maximum.

**Figure 11:**
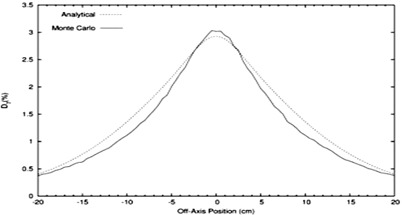
Comparison of the off‐axis X‐ray percent dose profiles for analytical and MC simulations of the 13 MeV beam. Doses are a percentage of central‐axis dose maximum. The simulations include X‐ray dose from accelerator exit window, primary foil, and secondary foil only. Plots are 2 cm downstream of isocenter.

Also, off‐axis X‐ray percent dose profiles, calculated at shallow depths by the analytical and MC simulations, agreed well. Expressed as a percent of the central‐axis dose maximum, the agreement was within 1.0% over the off‐axis range (−20cm, 20 cm) for 7 MeV to 20 MeV. Figure 11 shows a comparison plot of off‐axis X‐ray percent dose profiles for the analytical and MC simulations of the 13 MeV beam.

### B. Sample relative dose calculations

**Figure 12:**
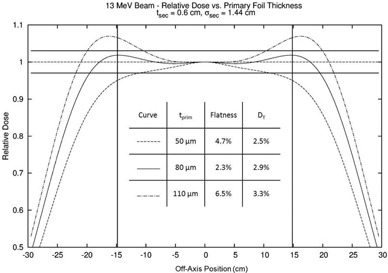
Comparison of total relative dose profiles for a 13 MeV beam calculated 2 cm beyond isocenter for tantalum primary foil thicknesses of 50, 80, and 110 μm and an aluminum Gaussian secondary foil with a maximum thickness of 0.6 cm and a sigma of 1.44 cm. The vertical lines represent the range of interest for flatness ($\pm \;14.6\;\text{cm},\;2\sqrt{2}\;\text{cm}$ inside the edges of the diagonal for a $25\times 25\;{\text{cm}}^{2}$ applicator). The horizontal lines represent a ±3% criterion for acceptable flatness.

One key benefit of using the real‐time dose simulator is that it gives the user the ability to gain a better understanding of how different calculation parameters affect the relative dose profile. The thickness of the primary foil is the main determining factor for the distribution of electron fluence that is incident on the secondary scattering foil and for X‐ray dose. Figure 12 demonstrates how the off‐axis total relative dose profile is affected by the thickness of the primary foil. All profiles were calculated 2 cm beyond isocenter for a beam with an accelerator energy of 13 MeV. For this simulation, the secondary foil is a 0.6 cm thick aluminum Gaussian foil with a sigma of 1.44 cm. As the figure shows, increasing the thickness of the primary foil from 50 to 110 μm increased the dose in the shoulders of the profile. This is due to the increased primary foil thickness increasing the relative number of electrons that scatter away from the central axis. Also shown in the Fig. 12 insert, the X‐ray dose increased with increasing primary foil thickness; doubling the primary foil thickness increased the X‐ray dose by 0.7%. Despite its small thickness, the high‐Z primary foil has a high radiative stopping power, resulting in its contributing significantly to the X‐ray dose generated by the dual scattering foil system.

**Figure 13:**
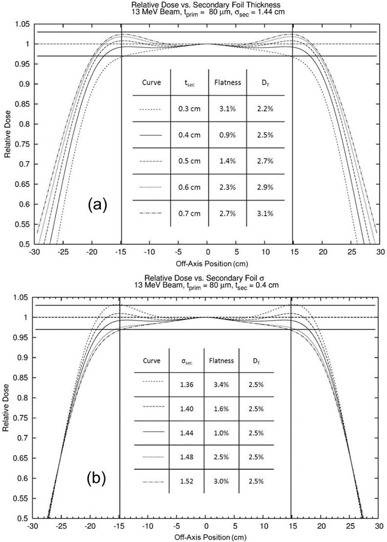
Comparisons of total relative dose profiles for a 13 MeV beam calculated 2 cm beyond isocenter with a tantalum primary foil thickness of 80 μm and an aluminum Gaussian secondary foil with (a) a sigma of 1.44 cm and varying thickness (0.3–0.7 cm) and (b) a thickness of 0.4 cm and varying sigma (1.36–1.56 cm). The vertical lines represent the range of interest for flatness ($\pm \;14.6\;\text{cm},\;2\sqrt{2}\;\text{cm}$ inside the edges of the diagonal for a $25\times 25\;{\text{cm}}^{2}$ applicator). The horizontal lines represent ±3% criterion for acceptable flatness.

The other main geometrical determiners of the off‐axis total relative dose profile are the thickness and sigma of the Gaussian shape of the secondary scattering foil. Figure 13 shows the off‐axis total relative dose profiles calculated 2 cm beyond isocenter for a variety of secondary aluminum foil thicknesses $\left(t_{\text{sec}}\right)$ and Gaussian sigmas $\left(\sigma_{\text{sec}}\right)$. The primary foil for each simulation was 80 μm thick and constructed of tantalum. In Fig. 13(a) the secondary foil sigma was constant (1.44 cm), while the thickness of the foil was varied (0.3–0.7 cm). As the secondary foil thickness increased, the overall width of the dose profile increased and the prominence of the off‐axis “horns” increased due to more of the electrons near the central axis being scattered to the edges. This effect is due to the central‐axis planar fluence per incident electron decreasing while the width of the distribution increases. Also shown in the Fig. 13(a) insert, $\%D_{\gamma ,\text{CAX}}$ increased approximately linearly with secondary foil thickness, doubling the secondary foil thickness increased the X‐ray dose by 0.7%, the same as seen for the primary foil. In Fig. 13(b), the secondary foil thickness was constant (0.4 cm), while the sigma of the foil was varied (1.36–1.52 cm). As sigma was increased, the magnitude of the horns decreased, while the width of the profile and $\%D_{\gamma ,\text{CAX}}$ remained relatively constant.

### C. Dual scattering foil design process

In addition to the educational utility of the dual scattering foil simulator, as illustrated in the previous section, it can be used as a design tool for a dual scattering foil system. We employed the use of objective profiles to design dual scattering foils for a range of accelerator energies (6–20 MeV). An objective profile is the ideal off‐axis total relative dose profile that the design procedure is attempting to produce. After designing a profile that matches the objective profile as closely as possible, a secondary check of the design configuration is necessary.

This secondary check can come either in the form of measuring the profile for the foil configuration or, more practically, simulating the design configuration using MC methods. By comparing the dose profile resulting from MC simulations with the one predicted by the analytical simulator, the objective profile can be slightly modified to yield a better prediction of foil design outcome, accounting for the physics not modeled by the dual foil simulator (e.g., large angle scatter and collimator scatter).

**Figure 14:**
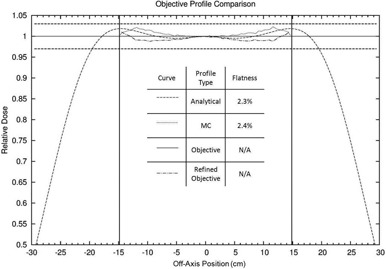
Comparison of total relative dose profiles for an optimized foil design for a 13 MeV beam. The dual foil configuration was designed for which the analytical profile closely matched the objective profile. The MC simulation profile for that configuration disagreed slightly with the analytical simulation profile. Therefore, the objective profile was refined, equaling the original objective profile (relative dose=1.0) plus the simulator calculated profile less the MC calculated dose profile. The foil configuration simulated was an 80 μm thick tantalum primary foil and a 0.6 cm thick aluminum Gaussian secondary foil with a sigma of 1.44 cm. The vertical lines represent the range of interest for flatness (±14.6cm (i.e., $2\sqrt{2}\;\text{cm}$ inside the edge of the diagonal) for a $25\times 25\;{\text{cm}}^{2}$ applicator). The horizontal lines represent ±3% criterion for acceptable flatness.

Figure 14 shows an example of the foil design process using a flat objective profile, $D_{\text{tot}}^{\text{obj}}(d=2\text{cm},\;r,\;E=13\;\text{MeV})=1.0$. The simulator produced a profile that closely matched the objective profile using a manually optimized dual scatting foil configuration (80 μm thick tantalum primary foil; 0.6 cm thick, 1.44 cm sigma aluminum secondary foil). As is evidenced by this plot, the MC calculated profile and the simulator calculated profile are slightly different. The analytical profile predicted a flatness of 2.3% and X‐ray dose of 2.9%, while the MC simulation predicted a flatness of 2.4% and an X‐ray dose of 3.2%. While the overall flatness prediction of the analytical simulator was reasonably accurate, it underpredicted the off‐axis MC relative dose by as much as 1%.

Future foil designs at this and nearby accelerator energies require modifying the objective profile. This refinement can be done by subtracting the difference between the MC simulated profile and the original analytic profile from the objective profile, $D_{\text{tot}}^{\text{obj},\text{ref}}(r)=D_{\text{tot}}^{\text{obj}}–[D_{\text{tot}}^{\text{MC}}(r)–D_{\text{tot}}^{\text{sim}}(r)]$. This process is done over the desired region of flatness (e.g., $2\sqrt{2}\;\text{cm}$ within the length of the diagonal of the largest available field size). Designing to the new objective profile will yield a more accurate prediction of the flatness of the design configuration. For a more in‐depth description and utilization of the objective profile design method, see LeBlanc.^(14)^

## IV. CONCLUSIONS

We have developed an analytical, real‐time, dual scattering foil simulator as an educational and design tool. This simulator has been shown to accurately predict: 1) MC‐simulated off‐axis electron relative dose profiles and off‐axis X‐ray percent dose profiles, and 2) measured central‐axis X‐ray percent dose. The real‐time nature and user‐friendly GUI of the simulator make it a unique and powerful educational tool for gaining a better understanding of the effects of a variety of dual foil parameters on off‐axis electron dose profiles and contamination X‐ray dose. The simulator has also demonstrated how to be a fast and efficient tool for designing dual scattering foil systems while greatly reducing the number on MC simulations required.

## ACKNOWLEDGMENTS

Portions of this research were conducted with high‐performance computational resources provided by Louisiana State University (http://www.hpc.lsu.edu).
